# Behavioral features and disorganization of oscillatory activity in C57BL/6J mice after acute low dose MK-801 administration

**DOI:** 10.3389/fnins.2022.1001869

**Published:** 2022-09-14

**Authors:** Keke Cui, Zhipeng Yu, Le Xu, Wangcong Jiang, Luwan Wang, Xiangqun Wang, Dandan Zou, Jiajie Gu, Feng Gao, Xiaoqing Zhang, Zhengchun Wang

**Affiliations:** ^1^Department of Pharmacology, Ningbo University School of Medicine, Ningbo, China; ^2^The Affiliated People’s Hospital of Ningbo University, Ningbo, China; ^3^Key Laboratory of Addiction Research of Zhejiang Province, Kang Ning Hospital, Ningbo, China

**Keywords:** MK-801, schizophrenia, N-methyl-D-aspartate receptor, local field potential, C57BL/6J

## Abstract

Low dose acute administration of *N*-methyl-D-aspartate receptor (NMDAR) antagonist MK-801 is widely used to model cognition impairments associated with schizophrenia (CIAS) in rodents. However, due to no unified standards for animal strain, dose, route of drug delivery, and the duration of administration, how different doses of MK-801 influence behavior and fundamental frequency bands of the local field potential (LFP) in cortical and subcortical brain regions without consistent conclusions. The optimal dose of MK-801 as a valid cognition impairers to model CIAS in C57BL/6J mice remains unclear. The current study characterizes the behavior and neural oscillation alterations induced by different low doses of MK-801 in medial prefrontal cortex (mPFC) and hippocampus CA1 of C57BL/6J mice. The results reveal that mice treated with 0.1 and 0.3 mg/kg MK-801 demonstrate increased locomotion and diminished prepulse inhibition (PPI), while not when treated with 0.05 mg/kg MK-801. We also find that MK-801 dose as low as 0.05 mg/kg can significantly diminishes spontaneous alteration during the Y-maze test. Additionally, the oscillation power in delta, theta, alpha, gamma and HFO bands of the LFP in mPFC and CA1 was potentiated by different dose levels of MK-801 administration. The current findings revealed that the observed sensitivity against spontaneous alteration impairment and neural oscillation at 0.05 mg/kg MK-801 suggest that 0.05 mg/kg will produce changes in CIAS-relevant behavior without overt changes in locomotion and sensorimotor processing in C57BL/6J mice.

## Introduction

Schizophrenia is one of the top 20 leading causes of disability (2018) that affects approximately 0.7% worldwide (McGrath et al., 2008; Moreno-Küstner et al., 2018). Economic burdens related to schizophrenia are as high as more than $150 billion annually in the United States (Cloutier et al., 2016; Jin and Mosweu, 2017). Schizophrenia treatment remains a major challenge, especially the associated animal models cannot totally reproduce the psychological factors of schizophrenia patients. Despite limitations existed in animal models of schizophrenia, they are potent investigative tools when used to model specific symptoms of a neuropsychiatric disorder. Acute administration of the *N*-methyl-D-aspartate receptor (NMDAR) noncompetitive antagonists (phencyclidine, MK-801 and ketamine) results in cognition impairments associated with schizophrenia, social interaction dysfunctions, lower thresholds of psychopathic behavior (Meltzer et al., 2013; Cadinu et al., 2018), and abnormal electrical activities of the brain in rodents and primates (Hunt and Kasicki, 2013). Particularly, MK-801 (Dizocilpine) is widely used as a pharmacological tool to evoke psychotic-like behaviors and disturbed local field potential (LFP) signal existed in several neuropsychiatric disorders (Hunt and Kasicki, 2013; Neill et al., 2014).

However, a close examination of literatures showed that the behavioral aberrations and the electrical activity alterations induced by different doses of MK-801 without unanimous definition. Firstly, the administration dose range of MK-801 was relatively narrow among various behavioral paradigms. For example, as is commonly done at the second half hour after drug injection, 0.15 mg/kg MK-801 increased locomotion activities, while the increment in locomotion was quite minimal with a single dose of 0.3 mg/kg (Wu et al., 2005); Additionally, MK-801 doses around 0.1 mg/kg enhanced locomotor activities, while 0.02 mg/kg MK-801 resulted in marked reduction of locomotion (Tang et al., 2006); stereotypy was not observed in animals given 0.05 mg/kg MK-801, while detected at 0.15 mg/kg (Wu et al., 2005). Secondly, in the NMDAR hypofunction model of schizophrenia, how electrical activities changed that might mediate the perceptual and cognitive impairments remained controversial. For example, with 0.1 mg/kg MK-801 administration, there were no changes in theta band power of motor/visual cortex (Phillips et al., 2012) as well as CA3 (Saunders et al., 2012), while decreased (Dimpfel and Spüler, 1990) or increased (Sebban et al., 2002) in frontal cortex. Moreover, variation in behaviors and electrical activities depending on the animal strain, gender of the subject, way of drug delivery and the timing of administration made it difficult to compare the behavioral and electrophysiological characteristics reported in different investigations.

Each mouse strain seems to show different dose-response relationships for behaviors and electrical activity induced by low dose of MK-801 (Mabunga et al., 2019), emphasizing the need for improvement in this area. The behavioral aberrations and neural electrical activity alterations induced by different doses of MK-801 in C57BL/6J mice have not been comprehensively quantified until now. C57BL/6J mouse is one of the most commonly used strains because it is robust, easy to replicate and frequently used in transgenic techniques. Therefore, the MK-801-induced behavioral aberrations such as hyperactivity, cognition impairment, and declined prepulse inhibition (PPI) as well as MK-801-evoked electrical activity alterations are characterized using 8–9 weeks old male C57BL/6J mice, which provide an experimental reference for future studies.

## Materials and methods

### Animals

Male C57BL/6J mice (7–8 weeks) were obtained from the Zhejiang Academy of medical Sciences (Hangzhou, Zhejiang, China) and habituated for at least 5 days under conditions with a constant temperature (22 ± 1°C) and humidity (50 ± 10%), a 12 h light/dark cycle, and free access to food and water. All procedures were carried out in accordance with the National Institutes of Health Guide for the Care and Use of Laboratory Animals, and were implemented according to the Animal Care and Use Committees of Ningbo University, China. Two batches of mice were used in the current study. The animal were about 8–9 weeks old when they were tested and they were run on behavioral tasks at least 1 week after animal shipment. In the open field test (OFT), PPI and LFP recordings, all animals were acclimated to the behavioral facility at least 1 h in advance.

### Drugs

The MK-801 powder (Abcam, England) was dissolved in 0.9% NaCl solution and administered intraperitoneally at a volume of 10 ml/kg of body weight, while the vehicle group received the same volume of saline. The dose selection (0.05, 0.1, and 0.3 mg/kg) for the MK-801 was based on previous reports (Adell et al., 2012; Hiyoshi et al., 2014; Torrisi et al., 2017; Cieślik et al., 2019; Mabunga et al., 2019) and our preliminary studies. All behavioral tests were started 30 min after MK-801 or saline were injected.

### Local field potential

#### Electrode implantation surgery

Mice were anesthetized with sodium pentobarbital (80 mg/kg, intraperitoneally) and fixed in a stereotaxic apparatus (Ruiwode, Shenzhen, China). After the skull was exposed, two 1 mm × 1 mm craniotomies were performed ipsilaterally, centered on areas of the mPFC (AP: +1.8; ML: +0.3; DV: −2.5 mm) and the CA1 (AP: −1.7; ML: +1.0; DV: −1.5 mm). The custom-made electrode with two channels was implanted into the mPFC and the CA1, respectively. Four stainless-steel screws were implanted into the skull with one doubling as the reference and ground electrode. The other screws together with dental acrylic (Hualun Medical Instrument Co., China) were used to fix the electrode onto the skull. The electrode-implanted mice were laid on a thermo-regulated blanket to facilitate anesthesia recovery and were then singly housed until electrophysiology recording (more than 1 week interval).

#### Local field potential recording

All the LFP recordings were performed during the light phase of the light/dark cycle. Cerebus 64-channel system (Blackrock microsystems) was used to amplified and filtered the LFP signals that were sampled at 1 kHz with a low-pass filter (<250 Hz). A baseline LFP was recorded for 30 min before MK-801 (0.05, 0.1, and 0.3 mg/kg) administration and LFP recording was continued for another 120 min. Each mouse was transferred to a square plexiglass chamber (40 cm × 40 cm × 40 cm) placed within an electrically shielded device. All LFP recordings were performed in mice under awake, quiet and unrestrained conditions. LFP recordings were repeated 2–4 times in each mouse after a more than 7 day interval for drug washout.

### Data analysis

All the LFP data were individually analyzed off-line with the analysis program NeuroExplorer (NexTechnologies, Colorado Springs, CO, United States) and custom-made Matlab programs. After digital filtering (0.1–200 Hz bandpass), a fast Fourier transform was performed for each 1 s epoch for a power spectrum analysis. Power and coherence spectra were calculated using 2,048-point fast Fourier transform and smoothed with 3-point Gaussian sliding window. For power spectral density, the power was normalized using logarithmic scale and the mean value of baseline recordings 30–60 min after administration. The LFP activities spanning across the 1–4, 6–8, 8–12, 30–60, 60–100, and 150–200 Hz band were defined as delta, theta, alpha, high gamma and HFO (oscillations), respectively. For the time course change of LFP power, the total power of frequency band was averaged for each 1 min bin and normalized with the mean value in the 30 min baseline recordings. The effect of the drug was evaluated by calculating the area under the curve (AUC) of LFP power 30–60 min after administration. The time windows to assess the dose response were selected based on the drug pharmacokinetic profiles (Hiyoshi et al., 2014).

### Behavioral tests

#### Open field test

Handling and acclimation of animals were conducted before any behavioral test. The locomotor and exploratory activity of the MK-801 or saline treated mice were measured by open field test (OFT). MK-801 or saline was injected 30 min before the OFT. Each mouse was gently placed in a square plexiglass chamber (40 cm × 40 cm × 40 cm) and allowed to freely explore the area for 30 min. The locomotor activity of four mice (0.05, 0.1, and 0.3 mg/kg and saline group, respectively) were monitored simultaneously at a time. All tests were recorded by an automated video-tracking system (Xiaoyi, Beijing, China) and analyzed off-line by Any-maze V5.33 (Stoelting, IL, United States).

#### Y-maze spontaneous alteration behavior test

Spontaneous alteration and exploration activities were measured with a Y-shaped maze with three white, opaque, plastic arms (40 cm long, 10 cm wide, and 15 cm high) at a 120° angle from each other. After introduction to the central intersection of the Y-maze, the animal was allowed to freely explore the three arms for 8 min. An arm entry was recorded when four paws of the mouse were within the arm zone and a spontaneous alternation was counted when three successive entries to different arms. The percentage of spontaneous alternations was defined as [total alternations/(total entries – 2)] × 100. Total arm entries and spontaneous alternations indicated the exploratory activity and cognitive function of the tested mice, respectively.

#### Prepulse inhibition test

**FIGURE 1:**
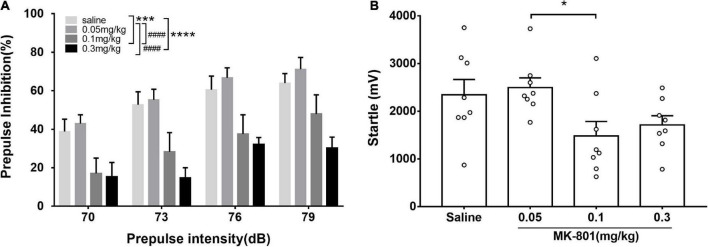
Acute MK-801 administration disrupted the prepulse inhibition of mice. PPI testing started after 30 min. drug injection. The prepulse inhibition **(A)** and amplitude of 120 dB responses **(B)** were analyzed. All values were expressed as mean ± SEM (*n* = 8 mice per group). *p < 0.05, ***p < 0.001, ****p < 0.0001 compared with the saline group and ^####^*p* < 0.0001 compared with the 0.3 mg/kg group as revealed by Tukey’s multiple comparisons test.

Prepulse inhibition (PPI) was used to measure sensory gating in animal. The PPI testing used here had been described in detail in previous publications (Valsamis and Schmid, 2011; Kobayashi et al., 2021; Si et al., 2021). Firstly, the mice was tenderly constrained in the holder during the whole measurement process. Following sufficient acclimation (defecation and urination ceased or considerably decreased in the holder), the startle response of the mice was detected using an accelerometer located underneath the holder. Each mouse underwent a startle habituation (5 min, 60 dB white noise, repeated five times) before PPI recording. The PPI measurement contained 50 trials which were presented in a pseudorandom order with 20–40 s inter-trial interval. Five types of stimuli were presented: the startle-alone stimulus (120 dB, 40 ms) and four types of PPI stimuli consisted a prepulse (70, 73, 76, or 79 dB, 40 ms) and a startle stimulus (120 dB, 40 ms) and the interval between the prepulse and the startle stimuli was 80 ms (Figure 1). The PPI for each prepulse intensity level was calculated as follows:

PPI(%)=(1-A_PPI/A_startle)×100%

Where A_PPI indicated the reaction amplitude of the mouse to each type of PPI stimulus, A_startle represented the reaction amplitude to the startle-alone stimulus.

### Statistical analysis

All data were displayed as means ± SEM. Statistical significance (*p* < 0.05) was assessed by the one-way ANOVA, two-way repeated measures ANOVA and unpaired *t*-test followed by Tukey’s *post-hoc* test using the GraphPad Prism Version 6.01 (CA, United States).

## Result

### MK-801 dose-dependently increased locomotor activity

**FIGURE 2:**
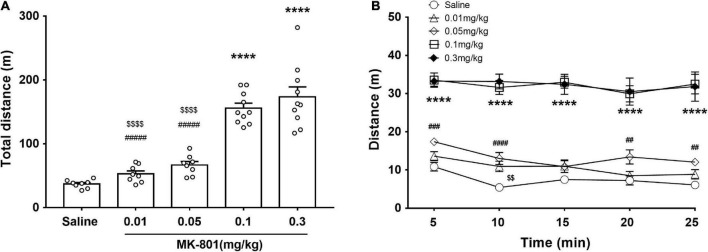
Effects of MK-801 injection on the locomotor activity of mice in the OFT. MK-801 (0.01, 0.05, 0.1, and 0.3 mg/kg) or 0.9% NaCl were injected intraperitoneally 30 min prior to the OFT. The total distance of mice moved **(A)** and their progression in a 5 min bin during the last 25 min in the OFT **(B)** were analyzed. All values were expressed as mean ± SEM (Saline: *n* = 8; MK-801, 0.01 mg/kg: *n* = 8; 0.05 mg/kg: *n* = 8; 0.1 mg/kg: *n* = 10; 0.3 mg/kg: *n* = 10). ****p < 0.0001 vs. the saline group, ^$$^*p* < 0.01, ^$$$$^*p* < 0.0001 vs. the 0.1 mg/kg group, and ^##^*p* < 0.01, ^###^*p* < 0.001, ^####^*p* < 0.0001 vs. the 0.3 mg/kg group as revealed by Tukey’s multiple comparisons test.

The effects of different doses of MK-801 on the locomotor activity were monitored by the open field test (OFT) (Figure 2). The total distance traveled shown and analyzed in Figure 2A [F(4, 39) = 45.47, *p* < 0.0001, one-way ANOVA, Tukey’s multiple comparisons test]. Compared with the saline group, both 0.1 mg/kg [F(4, 39) = 45.47, *p* < 0.0001] and 0.3 mg/kg [F(4, 39) = 45.47, *p* < 0.0001] MK-801 injections significantly increased the locomotor activity as revealed by longer distance moved in testing, while both 0.01 mg/kg [F(4, 39) = 45.47, *p* = 0.79] and 0.05 mg/kg [F(4, 39) = 45.47, *p* = 0.23] had no discernible effect on the locomotion (Figure 2A). Since the 0.01 mg/kg dose acted similar effect as to the 0.05 mg/kg, the 0.01 mg/kg was ruled out in the following experiment. Accordingly, their progression in a 5 min bin during the last 25 min in the OFT was showed in Figure 2B. Two-way repeated measures ANOVA revealed that there were significant main effects of time [F(4, 28) = 7.13, *p* = 0.004] and dose [F(4, 28) = 64.16, *p* < 0.0001] on PPI, while no interaction between them [F(16, 112) = 1.39, *p* = 0.17].

### MK-801 impaired spontaneous alteration

**FIGURE 3:**
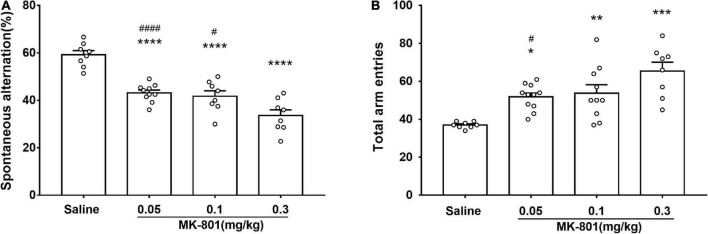
MK-801 administration resulted in declined spatial working memory of mice in Y-maze. Behavioral testing started following 30 min MK-801 or 0.9% NaCl injection. The percentage of spontaneous alternations **(A)** and the total number of arm entries **(B)** were analyzed. All values were expressed as mean ± SEM (Saline: *n* = 8; MK-801, 0.05 mg/kg: *n* = 10; 0.1 mg/kg: *n* = 10; 0.3 mg/kg: *n* = 8). *p < 0.05, **p < 0.01, ***p < 0.001, ****p < 0.0001 vs. the saline group and ^#^*p* < 0.05, ^####^*p* < 0.0001 vs. the 0.3 mg/kg group as revealed by Tukey’s multiple comparisons test.

The effects of acute MK-801 exposure (0.05, 0.1, and 0.3 mg/kg) on spontaneous alteration were assessed by the Y-maze test (Figure 3). Both the percentage of spontaneous alternations [Figure 3A; F(3, 30) = 29.4, *p* < 0.0001] and the total number of arm entries [Figure 3B, F(3, 33) = 10.34, *p* = 0.0192] in each MK-801-treated group were all changed compared with the saline group. Particularly, MK-801-treated group displayed decreased (0.05 mg/kg, *p* < 0.0001; 0.1 mg/kg, *p* < 0.0001; 0.3 mg/kg, *p* < 0.0001) the percentage of spontaneous alternations while and increased the total number of arm entries (0.05 mg/kg, *p* = 0.0192; 0.1 mg/kg, *p* = 0.0080; 0.3 mg/kg, *p* < 0.0001) compared with the control group, which indicated impaired spatial working memory of mice after MK-801 administration.

### MK-801 dose-dependently diminished prepulse inhibition

Prepulse inhibition was a paradigm for measuring the sensorimotor gating function which was widely used in evaluating animal models of schizophrenia. Animals in the four groups (0.05, 0.1, and 0.3 mg/kg and saline) were counterbalanced and four prepulse intensities (70, 73, 76, and 79 dB) were used in the PPI measurement. Two-way repeated measures ANOVA revealed that there were significant main effects of prepulse intensities [F(3, 84) = 6.50, *p* < 0.001] and groups [F(2, 84) = 18.49, *p* < 0.0001] on PPI, while no interaction between them [F(6, 84) = 0.33, *p* = 0.9201]. Particularly, compared with the control group, PPI in the 0.1 and 0.3 mg/kg MK-801 groups was significantly reduced (0.1 mg/kg, *p* = 0.0007; 0.3 mg/kg, *p* < 0.0001), while there was no difference in the 0.05 mg/kg MK-801 group (*p* > 0.9999). Furthermore, no significant differences (0.05 mg/kg, *p* = 0.9768; 0.1 mg/kg, *p* = 0.1148; 0.3 mg/kg, *p* = 0.3383) were observed in amplitudes of responses to the startle-alone stimulus (120 dB, 40 ms) between the saline and MK-801-treated mice. There was a statistical difference between 0.05 and 0.1 mg/kg MK-801 group (*p* = 0.0491).

### MK-801 dose-dependently increased the power of theta and high frequency oscillation band in the medial prefrontal cortex

**FIGURE 4:**
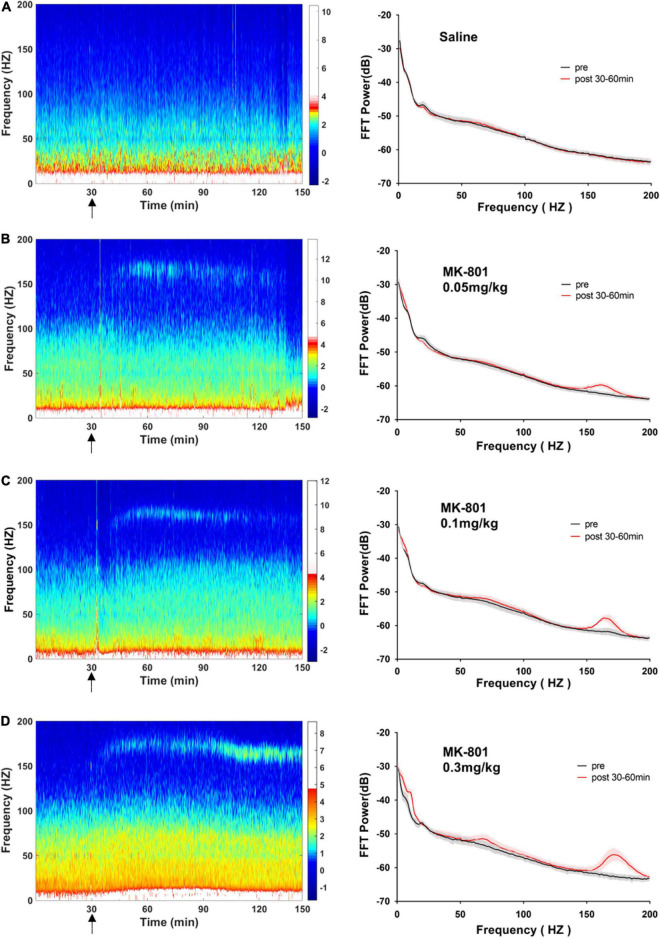
Effects of MK-801 on LFP in the prefrontal cortex. The *left panels* showed the heatmap for time courses of changes in LFP power. The *right panels* showed the averaged power spectrum density (PSD) in all tested frequency bands. The averaged LFP power in the saline group **(A)** and the 0.05 mg/kg MK-801 **(B)**, 0.1 mg/kg MK-801 **(C)**, and 0.3 mg/kg MK-801 **(D)** groups were displayed in the form of heatmap and PSD, respectively. The arrow indicated the moment of drug injection. Intracranial electrical signals collected from 4 to 8 mice per group and were recorded continuously before and after drug administration (Saline: *n* = 8; MK-801, 0.05 mg/kg: *n* = 8; 0.1 mg/kg: *n* = 5; 0.3 mg/kg: *n* = 4).

**FIGURE 5:**
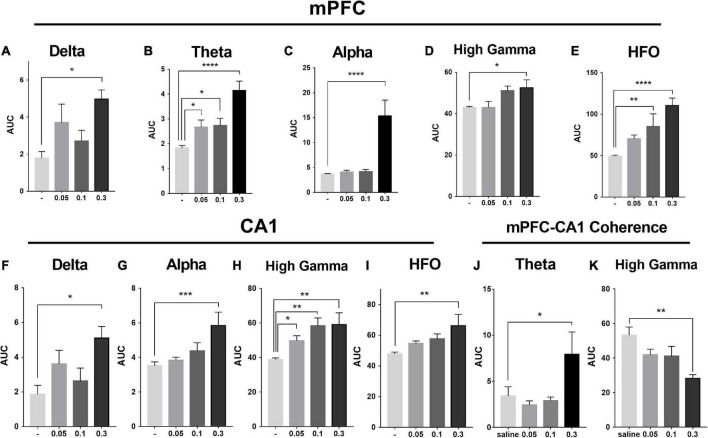
Effects of MK-801 on the area under the curve (AUC) of each LFP power in the prefrontal lobe and hippocampus. The up row displayed AUC values of the mPFC for 30–60 min. after drug administration in transitions of delta power (1–4 Hz; **A**), theta power (6–8 Hz; **B**), alpha power (8–12 Hz; **C**), high gamma power (60–100 Hz; **D**), and HFO power (150–200 Hz; **E**). The down row displayed AUC values of the CA1 for 30–60 min. after drug administration in transitions of delta power **(F)**, alpha power **(G)**, high gamma power **(H)**, and HFO power **(I)**. The data represented the mean ± SEM obtained from 4 to 8 animals per group. *, **, *** and **** indicated *p* < 0.05, *p* < 0.01, *p* < 0.001, *p* < 0.0001 compared to the vehicle. Theta **(J)** and gamma **(K)** power coherence between mPFC and CA1 under saline and MK-801 (0.05, 0.1, and 0.3 mg/kg) condition during 30–60 min. post-dosing.

As showed in the Figure 4 and Table 1, MK-801 dose-dependently altered the power of LFP in the mPFC. One way ANOVA revealed that 0.05 mg/kg MK-801 increased [F(3, 20) = 13.43, *p* = 0.0336] the power of the theta band (30–60 min. post-injection); 0.1 mg/kg MK-801 increased both the theta [F(3, 20) = 13.43, *p* = 0.0403] and high frequency oscillation (HFO) [F(3, 20) = 11.18, *p* = 0.0062]; 0.3 mg/kg MK-801 increased the delta [F(3, 19) = 3.335, *p* = 0.0212], theta [F(3, 20) = 13.43, *p* < 0.0001], alpha [F(3, 21) = 22.72, *p* < 0.0001], gamma [F(3, 21) = 4.033, *p* = 0.0206] and HFO [F(3, 20) = 11.18, *p* < 0.0001; Figure 5 up].

**TABLE 1:** Effects of MK-801 on the delta, theta, alpha, gamma, and high frequency oscillation (HFO) recorded in the prefrontal cortex and hippocampus.

Frequency (HZ)					MK-801Dose (mg/kg)	Structure
Delta	Theta	Alpha	High gamma	HFO		
		
1–4	6–8	8–12	60–100	150–200		
–	↑	–	–	–	0.05	mPFC
–	↑	–	–	**↑↑**	0.1	
↑	↑↑↑↑	↑↑↑↑	↑	**↑↑↑↑**	0.3	
–	–	–	↑	–	0.05	CA1
–	–	–	↑↑	–	0.1	
↑	–	↑↑↑	↑↑	↑↑	0.3	
–	↑	–	↓↓	–	0.3	mPFC-CA1
						coherence

– means there is no changes. ↑, ↑↑, ↑↑↑, and ↑↑↑↑ represent enhancement (*p* < 0.05, *p* < 0.01, *p* < 0.001 and *p* < 0.0001, respectively) in power. ↓↓ represents a decrease (*p* < 0.01) in power. Hz, Hertz; HFO, high frequency oscillations; CA1, CA1 field of the hippocampus; mPFC, medial prefrontal cortex.

### MK-801 increased the power of high gamma band in the CA1

**FIGURE 6:**
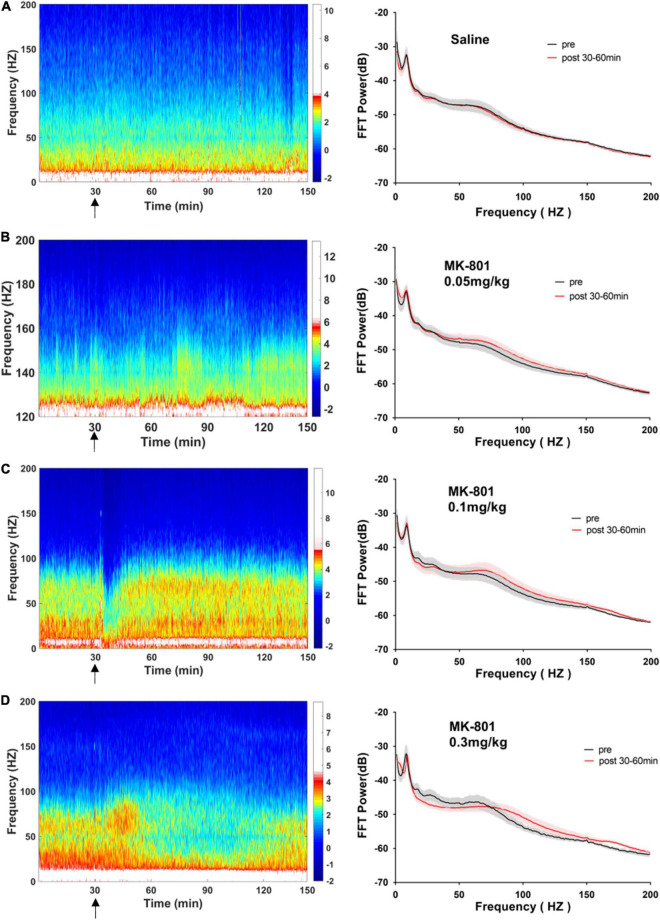
Effects of MK-801 on LFP in the CA1 of hippocampus. The same format as Figure 4.

As showed in the Figure 6 and Table 1, the power of gamma band in the CA1 was potentiated after MK-801 injection [F(3, 21) = 7.803, 0.0.5 mg/kg, *p* = 0.0450; 0.1 mg/kg, *p* = 0.0016; 0.3 mg/kg, *p* = 0.0022]. The power of the delta [F(3, 19) = 3.945, *p* = 0.0109], alpha [F(3, 21) = 6.737, *p* = 0.0009] and HFO [F(3, 21) = 6.028, *p* = 0.0013] band were increased after 0.3 mg/kg MK-801 administration. Additionally, the coherence of mPFC and CA1 was decreased in the power of high gamma frequency after 0.3 mg/kg MK-801 injection [F(3, 21) = 4.741, *p* = 0.0037; Figure 5 bottom].

## Discussion

Although several previous studies have confirmed MK-801 acute exposure as a valid model for schizophrenia, there was no consensus on dose-administration. A valid cognition impairer was defined as a compound that impaired learning and/or memory function without necessarily affecting locomotion and sensorimotor processing (van der Staay et al., 2011). Previous reports based on disrupted cognition performance of MK-801-treated rat suggested that 0.1 mg/kg MK-801 appeared to impair learning and/or memory processes without inducing adverse side effects such as sensorimotor impairments and/or signs of intoxication (van der Staay et al., 2011). Doses of MK801 of 0.075 and 0.15 mg/kg did not affect the accuracy of C57BL/6J mice in the operant-based two-choice visual discrimination task (Bitanihirwe et al., 2011). Still, a study used CD-1 mice to recapitulate behavioral impairments following acute administration of MK-801 observed that 0.1 mg/kg MK-801 diminished spontaneous alteration with no effects on total arm entries during the Y-maze test (Mabunga et al., 2019). Wu et al. (2005) also found that the dose of 0.05 mg/kg did not alter motor activities in C57BL/6J mice half an hour after MK-801 injection, while there were no experiments conducted to probe the cognitive function. It seems that the dose of MK-801 that evoked behavioral aberrations varied among experiments and across species. The current study provided evidence that low dose MK-801 administration induced neuropsychiatric behavioral features and evoked aberrations in rhythmic patterns of neural activity. Specifically, MK-801 dose-dependently increased locomotor activities, impaired spontaneous alteration and diminished PPI. Additionally, the neural oscillation power in local field potential (LFP) was potentiated on different dose levels following MK-801 administration, which was revealed by delta (1–4 Hz), theta (6–8 Hz), alpha (8–12 Hz), gamma (60–100 Hz), or HFO (150–200 Hz) band power in both mPFC and CA1, respectively. Particularly, 0.1 mg/kg MK-801 administered i.p. into C57BL/6J not only impaired spontaneous alteration in Y-maze, but also increased total arm entries, elevated locomotor activities in OFT and disturbed sensory gating in PPI measurement. Additionally, 0.05 mg/kg MK-801 effectively impaired spontaneous alternation in Y-maze but did not change the locomotion in OFT and spread no effect on PPI. The current experimental study found that acute administration of 0.05 mg/kg of MK-801 impaired spontaneous alternation in the Y-maze without affecting locomotion in C57BL/6J mice, which provided a reference for future investigations.

It is worth noting that although there was no statistically significant difference in the amplitude of startle response (A_startle) between the 0.1 and 0.3 mg/kg MK-801 groups compared with the saline group, there a trend toward a decrease. While there was a statistical difference between 0.05 and 0.1 mg/kg MK-801 group (*p* = 0.0491). To rule out the effect of startle reactivity on PPI levels in C57BL/6J mice (Shoji and Miyakawa, 2018), the reaction amplitude of the PPI (A_PPI) was reanalyzed. Compared with the saline group, the A_PPI was significantly increased in both the 0.1 and 0.3 mg/kg MK-801 groups [Two-way repeated measures ANOVA, F(3, 21) = 76.69, *p* < 0.0001]. It is revealed that the declined PPI after 0.1 and 0.3 mg/kg MK-801 administration was due to A_PPI.

The LFP activities spanning across different frequency band were differently altered following different doses of MK-801 treatment. Specifically, delta band power in mPFC was increased by 0.3 mg/kg MK-801; theta band power in mPFC was increased by all tested MK-801 doses; alpha band power in both mPFC and CA1 was potentiated by 0.3 mg/kg MK-801; gamma band power in mPFC was increased by 0.3 mg/kg MK-801, and gamma band power in CA1 was potentiated by all tested MK-801 doses; HFO in mPFC was increased by 0.1 mg/kg MK-801 and 0.3 mg/kg MK-801, and HFO in mPFC was increased by 0.3 mg/kg MK-801 (Table 1). One of the most concerned was the gamma oscillations, rhythmic patterns of electrical activities (60–100 Hz) observed in the LFP, owing to their association with perceptual and cognitive processes. In the current study, 0.3 mg/kg MK-801 induced an increment in gamma power of mPFC, and gamma power in CA1 was potentiated by all tested MK-801 doses. Those findings were consistent with previous results that MK-801 triggered enhanced power in spontaneous gamma oscillations in rodents (Carlén et al., 2012; Molina et al., 2014; Hudson et al., 2020). It had been reported that inhibitory parvalbumin (PV) interneurons played an important role in the coordinated interaction of excitation and inhibition that underlies the emergence of gamma oscillations (Buzsáki and Wang, 2012). Particularly, the NMDAR (*N*-methyl-D-aspartate receptor) in PV interneurons had been demonstrated to be critical for expression of normal gamma rhythms (Carlén et al., 2012; Picard et al., 2019; Hudson et al., 2020). Therefore, the PV interneurons might the central targets of NMDAR antagonist. It was worth noting that a recent study reported that pharmacologically induced enhancement of broadband gamma power was distinct from the increased spontaneous broadband caused by PV neuron dysfunction (Guyon et al., 2021). That was, the spontaneous gamma oscillations that were measured via a power increase across a wide frequency range at baseline or task-free conditions might be a reflection of increases in the overall level of circuit activities rather than of rhythmic neural activities that was synchronized across neurons (Sohal and Rubenstein, 2019). In addition, ketamine, another NMDAR antagonist, had been showed to result in a desynchronized state and enhanced broadband gamma power whether systemic administration (Mahdavi et al., 2020) or local application (Guyon et al., 2021). Given ketamine-treated LFP changes were found to be related to ketamine-induced alterations in the thalamic reticular nucleus and thalamocortical networks (Mahdavi et al., 2020), the enhanced gamma power might be similar to the effect of systemic ketamine of thalamic networks (Mahdavi et al., 2020). Prefrontal lobe and hippocampus are important brain regions related to cognitive function. Converging lines of evidence from cognitive assessment, multimodal brain imaging, postmortem studies, and electrophysiological studies point to PFC and hippocampal dysfunction in schizophrenia. NMDAr antagonists produce cognitive impairment that may result from the effect of these drugs on gamma oscillatory activity (Lewis et al., 2005). Functional connectivity between cortical and subcortical brain areas plays an important role in cognitive function. Reduced functional connectivity between hippocampal and cortical regions is associated with reduced hippocampal glutamate levels in patients with psychiatric disorders (Nelson et al., 2022). The current results consistently shows that the high gamma frequency coherence of CA1-PFC in mice decreases after acute low dose MK-801 administration. These results suggest that an alteration in the relationship between glutamate and functional connectivity may disrupt the dynamic of major neural networks.

Most often, researchers interpret NMDAR antagonist-induced behavior aberrations and neural activities deficits based on the conclusion that NMDAR antagonists preferentially regulated the firing rate of cortical inhibitory interneurons and the excitatory in pyramidal neuron caused by a net disinhibition was indirect (Homayoun and Moghaddam, 2007; Seamans, 2008; Coyle, 2012). More specifically, the sustained firing rate potentiation in the majority of PFC neurons produced by MK-801 could be sorted into random spike activities and burst activities (Jackson et al., 2004). The disrupted PFC function caused by MK-801 administration was due to an increase in the number of randomly distributed single spikes and simultaneous a profound reduction in burst activities (Jackson et al., 2004). By genetic ablation of NMDRs in corticolimbic interneurons, mutant mice showed a wide range of aberrant behaviors, including deficits in cognition and PPI, novelty-induced hyperlocomotion, and anxiety-like behaviors (Belforte et al., 2010). However, NMDAR ablation specifically in PV interneurons displayed largely normal behaviors except for selective cognitive impairments, including deficits in habituation, working memory and associative learning (Carlén et al., 2012). A latest study updated the notion of schizophrenia-related PV-specific NMDAR hypofunction by mute mice with NMDAR knockout from PV interneurons, which sensitized to schizophrenia-related deficits induced by MK-801 (Bygrave et al., 2016). These results suggested a model where NMDAR hypofunction in multiple cell types might contribute to the disease. A recent study reported that NMDAR on PV-positive interneurons and pyramidal neurons both contributed to MK-801 induced gamma oscillatory disturbances and engaged complex relationships with behavior (Hudson et al., 2020). Both acute and chronic administration of NMDA receptor antagonists are classic models of schizophrenia. Studies have shown that acute and sub-chronic administration of PCP in perinatal rats results in different patterns of neurodegeneration (Anastasio and Johnson, 2008). Compared with acute PCP administration, sub-chronic treatment produces a reduction in glucose utilization and blood flow in the prefrontal cortex (hypofrontality),which is more relevant modeling the non-psychotic symptoms of schizophrenia (Pratt et al., 2008). The persistent nature of effects produced by sub-chronic PCP withdrawal is one of the reasons that acute administration of PCP is more stable than the schizophrenia symptoms mimicked by chronic administration (Lydall et al., 2010; Fellini et al., 2014).

The potential linking between the behavioral findings and the electrophysiological results were still inconsistent. A single injection of noncompetitive NMDAR antagonists induces psychotic symptoms (including hallucinations), cognitive impairment and exacerbates symptoms in patients with schizophrenia (Adler et al., 1998). The NMDAR hypofunction-related pathophysiological cortical gamma oscillations are accompanied by abnormal behavior, including hyperlocomotion and ataxia (Ho et al., 2004; Jeon et al., 2007). Studies of cortical local field potentials evoked by MK-801 under different anesthesia conditions found that abnormal gamma oscillations were not dependent on hyperlocomotion-related brain state or conscious sensorimotor processing (Hakami et al., 2009). This was again verified in our experiments, where increased exercise in the 0.1 mg/kg group was not accompanied by a significant increase in prefrontal gamma oscillations. Additionally, ketamine and MK-801 increased sustained gamma power and decreased evoked gamma power, both of which are related to disrupted sensorimotor gating (Jones et al., 2014). Previous studies have shown that ketamine-induced changes in GBO power in the PFC are significantly associated with sensory gating (Qi et al., 2018). In our study, 0.3 mg/kg Mk-801 increased prefrontal gamma oscillation and impaired sensory gating function in mice. 0.1 mg/kg MK-801 impaired sensory gating, while, had no effect on prefrontal gamma oscillations. This may suggest that sensory gating function is regulated by multiple factors. Theta oscillations convey hippocampal inputs to the PFC and simultaneously synchronize the activity of these two regions during memory, learning and other cognitive tasks (Soltani Zangbar et al., 2020). The spontaneous alternating decrease of 0.05 mg/kg MK-801 may be related to the decrease of theta band in prefrontal lobe.

It is worthy to be pointed out that there could be local or institution-specific conditions that could potentially affect sensitivity to MK-801 considering animal husbandry choices, whether animals are bred in-house or ordered from a breeder, environmental factors at the testing site (e.g., sounds, construction) and the age of the testing animals. All those factors could lead to different effects at different testing sites, particularly if such factors interact with developmental sensitivity periods. Since the study was conducted at a single test site, we cannot totally rule out these potential factors. Therefore, it is difficult to tell from current findings whether C57BL/6J mice do have a higher sensitivity to MK-801. A meta-analysis or comparison of behavioral findings between C57BL/6J mice and other rodents across multiple sites were required to reveal a more definite conclusion.

In conclusion, considering the acute effects, high face and construct validity of pharmacological NMDAR antagonism model (Corbett et al., 1995; Tsai and Coyle, 2002; Coyle, 2006; Frohlich and Van Horn, 2014), MK-801 is still widely utilized as a model for symptoms observed in schizophrenia (Ranson et al., 2019). Pharmacological NMDAR antagonism presents a powerful experimental model in which to recapitulate of perceptual disturbances and cognition deficits in psychosis, while the neural mechanisms underlie those neuropsychiatric behavioral features have not been totally elucidated. Further studies will be required to confirm how MK-801 targets the NMDAR in different types of fast-spiking interneurons in different brain area.

## Data availability statement

The original contributions presented in this study are included in the article/Supplementary material, further inquiries can be directed to the corresponding author.

## Ethics statement

This animal study was reviewed and approved by Animal Care and Use Committees of Ningbo University, China.

## Author contributions

ZW initiated and designed this study. KC performed the electrophysiology recordings and analyzed the data. KC and LW performed the behavioral test. ZW, KC, WJ, XW, DZ, FG, and XZ wrote the manuscript. All authors contributed to the article and approved the submitted version.

## References

[B1] Adell A. Jiménez-Sánchez L. López-Gil X. Romón T. (2012). Is the acute NMDA receptor hypofunction a valid model of schizophrenia? *Schizophr. Bull.* 38 9–14. 10.1093/schbul/sbr133 21965469 PMC3245580

[B2] Adler C. M. Goldberg T. E. Malhotra A. K. Pickar D. Breier A. (1998). Effects of ketamine on thought disorder, working memory, and semantic memory in healthy volunteers. *Biol. Psychiatry* 43 811–816. 10.1016/s0006-3223(97)00556-8 9611670

[B3] Anastasio N. C. Johnson K. M. (2008). Differential regulation of the NMDA receptor by acute and sub-chronic phencyclidine administration in the developing rat. *J. Neurochem.* 104 1210–1218. 10.1111/j.1471-4159.2007.05047.x 17995927

[B4] Belforte J. E. Zsiros V. Sklar E. R. Jiang Z. Yu G. Li Y. (2010). Postnatal NMDA receptor ablation in corticolimbic interneurons confers schizophrenia-like phenotypes. *Nat. Neurosci.* 13 76–83. 10.1038/nn.2447 19915563 PMC2797836

[B5] Bitanihirwe B. K. Dubroqua S. Singer P. Feldon J. Yee B. K. (2011). Sensorimotor gating and vigilance-dependent choice accuracy: a within-subject correlative analysis in wild-type C57BL/6 mice. *Behav. Brain Res.* 217 178–187. 10.1016/j.bbr.2010.10.021 20974191

[B6] Buzsáki G. Wang X. J. (2012). Mechanisms of gamma oscillations. *Annu. Rev. Neurosci.* 35 203–225. 10.1146/annurev-neuro-062111-150444 22443509 PMC4049541

[B7] Bygrave A. M. Masiulis S. Nicholson E. Berkemann M. Barkus C. Sprengel R. (2016). Knockout of NMDA-receptors from parvalbumin interneurons sensitizes to schizophrenia-related deficits induced by MK-801. *Transl. Psychiatry* 6:e778. 10.1038/tp.2016.44 27070406 PMC4872402

[B8] Cadinu D. Grayson B. Podda G. Harte M. K. Doostdar N. Neill J. C. (2018). NMDA receptor antagonist rodent models for cognition in schizophrenia and identification of novel drug treatments, an update. *Neuropharmacology* 142 41–62. 10.1016/j.neuropharm.2017.11.045 29196183

[B9] Carlén M. Meletis K. Siegle J. H. Cardin J. A. Futai K. Vierling-Claassen D. (2012). A critical role for NMDA receptors in parvalbumin interneurons for gamma rhythm induction and behavior. *Mol. Psychiatry* 17 537–548. 10.1038/mp.2011.31 21468034 PMC3335079

[B10] Cieślik P. Radulska A. Pelikant-Małecka I. Płoska A. Kalinowski L. Wierońska J. M. (2019). Reversal of MK-801-induced disruptions in social interactions and working memory with simultaneous administration of LY487379 and VU152100 in mice. *Int. J. Mol. Sci.* 20:2781. 10.3390/ijms20112781 31174329 PMC6600181

[B11] Cloutier M. Aigbogun M. S. Guerin A. Nitulescu R. Ramanakumar A. V. Kamat S. A. (2016). The economic burden of schizophrenia in the United States in 2013. *J. Clin. Psychiatry* 77 764–771. 10.4088/JCP.15m10278 27135986

[B12] Corbett R. Camacho F. Woods A. T. Kerman L. L. Fishkin R. J. Brooks K. (1995). Antipsychotic agents antagonize non-competitive N-methyl-D-aspartate antagonist-induced behaviors. *Psychopharmacology* 120 67–74. 10.1007/bf02246146 7480537

[B13] Coyle J. T. (2006). Glutamate and schizophrenia: beyond the dopamine hypothesis. *Cell. Mol. Neurobiol.* 26 365–384. 10.1007/s10571-006-9062-8 16773445 PMC11881825

[B14] Coyle J. T. (2012). NMDA receptor and schizophrenia: a brief history. *Schizophr. Bull.* 38 920–926. 10.1093/schbul/sbs076 22987850 PMC3446237

[B15] Dimpfel W. Spüler M. (1990). Dizocilpine (MK-801), ketamine and phencyclidine: low doses affect brain field potentials in the freely moving rat in the same way as activation of dopaminergic transmission. *Psychopharmacology* 101 317–323. 10.1007/bf02244048 2163537

[B16] Fellini L. Kumar G. Gibbs S. Steckler T. Talpos J. (2014). Re-evaluating the PCP challenge as a pre-clinical model of impaired cognitive flexibility in schizophrenia. *Eur. Neuropsychopharmacol.* 24 1836–1849. 10.1016/j.euroneuro.2014.08.012 25300235

[B17] Frohlich J. Van Horn J. D. (2014). Reviewing the ketamine model for schizophrenia. *J. Psychopharmacol.* 28 287–302. 10.1177/0269881113512909 24257811 PMC4133098

[B18] Guyon N. Zacharias L. R. Fermino de Oliveira E. Kim H. Leite J. P. Lopes-Aguiar C. (2021). Network asynchrony underlying increased broadband gamma power. *J. Neurosci.* 41 2944–2963. 10.1523/jneurosci.2250-20.2021 33593859 PMC8018896

[B19] Hakami T. Jones N. C. Tolmacheva E. A. Gaudias J. Chaumont J. Salzberg M. (2009). NMDA receptor hypofunction leads to generalized and persistent aberrant gamma oscillations independent of hyperlocomotion and the state of consciousness. *PLoS One* 4:e6755. 10.1371/journal.pone.0006755 19707548 PMC2727800

[B20] Hiyoshi T. Kambe D. Karasawa J. Chaki S. (2014). Differential effects of NMDA receptor antagonists at lower and higher doses on basal gamma band oscillation power in rat cortical electroencephalograms. *Neuropharmacology* 85 384–396. 10.1016/j.neuropharm.2014.05.037 24907590

[B21] Ho B. C. Mola C. Andreasen N. C. (2004). Cerebellar dysfunction in neuroleptic naive schizophrenia patients: clinical, cognitive, and neuroanatomic correlates of cerebellar neurologic signs. *Biol. Psychiatry* 55 1146–1153. 10.1016/j.biopsych.2004.02.020 15184033

[B22] Homayoun H. Moghaddam B. (2007). NMDA receptor hypofunction produces opposite effects on prefrontal cortex interneurons and pyramidal neurons. *J. Neurosci.* 27 11496–11500. 10.1523/jneurosci.2213-07.2007 17959792 PMC2954603

[B23] Hudson M. R. Sokolenko E. O’Brien T. J. Jones N. C. (2020). NMDA receptors on parvalbumin-positive interneurons and pyramidal neurons both contribute to MK-801 induced gamma oscillatory disturbances: complex relationships with behaviour. *Neurobiol. Dis.* 134:104625. 10.1016/j.nbd.2019.104625 31786371

[B24] Hunt M. J. Kasicki S. (2013). A systematic review of the effects of NMDA receptor antagonists on oscillatory activity recorded in vivo. *J. Psychopharmacol.* 27 972–986. 10.1177/0269881113495117 23863924

[B25] Jackson M. E. Homayoun H. Moghaddam B. (2004). NMDA receptor hypofunction produces concomitant firing rate potentiation and burst activity reduction in the prefrontal cortex. *Proc. Natl. Acad. Sci. U.S.A.* 101 8467–8472. 10.1073/pnas.0308455101 15159546 PMC420417

[B26] Jeon H. J. Cho M. J. Cho S. J. Kim S. U. Park S. K. Kwon J. S. (2007). Quantitative analysis of ataxic gait in patients with schizophrenia: the influence of age and visual control. *Psychiatry Res.* 152 155–164. 10.1016/j.psychres.2006.09.001 17512059

[B27] Jin H. Mosweu I. (2017). The societal cost of schizophrenia: a systematic review. *Pharmacoeconomics* 35 25–42. 10.1007/s40273-016-0444-6 27557994

[B28] Jones N. C. Anderson P. Rind G. Sullivan C. van den Buuse M. O’Brien T. J. (2014). Effects of aberrant gamma frequency oscillations on prepulse inhibition. *Int. J. Neuropsychopharmacol.* 17 1671–1681. 10.1017/s1461145714000492 24832766

[B29] Kobayashi Y. Inaba H. Iwakura Y. Namba H. Sotoyama H. Murata Y. (2021). Inter-breeder differences in prepulse inhibition deficits of C57BL/6J mice in a maternal immune activation model. *Neuropsychopharmacol. Rep.* 41 416–421. 10.1002/npr2.12178 34043885 PMC8411318

[B30] Lewis D. A. Hashimoto T. Volk D. W. (2005). Cortical inhibitory neurons and schizophrenia. *Nat. Rev. Neurosci.* 6 312–324. 10.1038/nrn1648 15803162

[B31] Lydall E. S. Gilmour G. Dwyer D. M. (2010). Analysis of licking microstructure provides no evidence for a reduction in reward value following acute or sub-chronic phencyclidine administration. *Psychopharmacology* 209 153–162. 10.1007/s00213-010-1779-x 20145910

[B32] Mabunga D. F. N. Park D. Ryu O. Valencia S. T. Adil K. J. L. Kim S. (2019). Recapitulation of neuropsychiatric behavioral features in mice using acute low-dose MK-801 administration. *Exp. Neurobiol.* 28 697–708. 10.5607/en.2019.28.6.697 31902157 PMC6946115

[B33] Mahdavi A. Qin Y. Aubry A. S. Cornec D. Kulikova S. Pinault D. (2020). A single psychotomimetic dose of ketamine decreases thalamocortical spindles and delta oscillations in the sedated rat. *Schizophr. Res.* 222 362–374. 10.1016/j.schres.2020.04.029 32507548

[B34] McGrath J. Saha S. Chant D. Welham J. (2008). Schizophrenia: a concise overview of incidence, prevalence, and mortality. *Epidemiol. Rev.* 30 67–76. 10.1093/epirev/mxn001 18480098

[B35] Meltzer H. Y. Rajagopal L. Huang M. Oyamada Y. Kwon S. Horiguchi M. (2013). Translating the N-methyl-D-aspartate receptor antagonist model of schizophrenia to treatments for cognitive impairment in schizophrenia. *Int. J. Neuropsychopharmacol.* 16 2181–2194. 10.1017/s1461145713000928 24099265

[B36] Molina L. A. Skelin I. Gruber A. J. (2014). Acute NMDA receptor antagonism disrupts synchronization of action potential firing in rat prefrontal cortex. *PLoS One* 9:e85842. 10.1371/journal.pone.0085842 24465743 PMC3895008

[B37] Moreno-Küstner B. Martín C. Pastor L. (2018). Prevalence of psychotic disorders and its association with methodological issues. A systematic review and meta-analyses. *PLoS One* 13:e0195687. 10.1371/journal.pone.0195687 29649252 PMC5896987

[B38] Neill J. C. Harte M. K. Haddad P. M. Lydall E. S. Dwyer D. M. (2014). Acute and chronic effects of NMDA receptor antagonists in rodents, relevance to negative symptoms of schizophrenia: a translational link to humans. *Eur. Neuropsychopharmacol.* 24 822–835. 10.1016/j.euroneuro.2013.09.011 24287012

[B39] Nelson E. A. Kraguljac N. V. Maximo J. O. Briend F. Armstrong W. Ver Hoef L. W. (2022). Hippocampal dysconnectivity and altered glutamatergic modulation of the default mode network: a combined resting-state connectivity and magnetic resonance spectroscopy study in schizophrenia. *Biol. Psychiatry Cogn. Neurosci. Neuroimaging* 7 108–118. 10.1016/j.bpsc.2020.04.014 32684484 PMC7904096

[B40] Phillips K. G. Cotel M. C. McCarthy A. P. Edgar D. M. Tricklebank M. O’Neill M. J. (2012). Differential effects of NMDA antagonists on high frequency and gamma EEG oscillations in a neurodevelopmental model of schizophrenia. *Neuropharmacology* 62 1359–1370. 10.1016/j.neuropharm.2011.04.006 21521646

[B41] Picard N. Takesian A. E. Fagiolini M. Hensch T. K. (2019). NMDA 2A receptors in parvalbumin cells mediate sex-specific rapid ketamine response on cortical activity. *Mol. Psychiatry* 24 828–838. 10.1038/s41380-018-0341-9 30696941 PMC6756203

[B42] Pratt J. A. Winchester C. Egerton A. Cochran S. M. Morris B. J. (2008). Modelling prefrontal cortex deficits in schizophrenia: implications for treatment. *Br. J. Pharmacol.* 153 (Suppl. 1) S465–S470. 10.1038/bjp.2008.24 18311160 PMC2268056

[B43] Qi R. Li J. Wu X. Geng X. Chen N. Yu H. (2018). Effects of ketamine on basal gamma band oscillation and sensory gating in prefrontal cortex of awake rats. *Neurosci. Bull.* 34 457–464. 10.1007/s12264-018-0208-8 29380249 PMC5960446

[B44] Ranson A. Broom E. Powell A. Chen F. Major G. Hall J. (2019). Top-down suppression of sensory cortex in an NMDAR hypofunction model of psychosis. *Schizophr. Bull.* 45 1349–1357. 10.1093/schbul/sby190 30945745 PMC6811829

[B45] Saunders J. A. Gandal M. J. Siegel S. J. (2012). NMDA antagonists recreate signal-to-noise ratio and timing perturbations present in schizophrenia. *Neurobiol. Dis.* 46 93–100. 10.1016/j.nbd.2011.12.049 22245663 PMC4161042

[B46] Seamans J. (2008). Losing inhibition with ketamine. *Nat. Chem. Biol.* 4 91–93. 10.1038/nchembio0208-91 18202677

[B47] Sebban C. Tesolin-Decros B. Ciprian-Ollivier J. Perret L. Spedding M. (2002). Effects of phencyclidine (PCP) and MK 801 on the EEGq in the prefrontal cortex of conscious rats; antagonism by clozapine, and antagonists of AMPA-, alpha(1)- and 5-HT(2A)-receptors. *Br. J. Pharmacol.* 135 65–78. 10.1038/sj.bjp.0704451 11786481 PMC1573114

[B48] Shoji H. Miyakawa T. (2018). Relationships between the acoustic startle response and prepulse inhibition in C57BL/6J mice: A large-scale meta-analytic study. *Mol. Brain* 11:42. 10.1186/s13041-018-0382-7 30001725 PMC6044095

[B49] Si W. Liu X. Pape H. C. Reinscheid R. K. (2021). Neuropeptide S-Mediated modulation of prepulse inhibition depends on age, gender, stimulus-timing, and attention. *Pharmaceuticals* 14:489. 10.3390/ph14050489 34065431 PMC8160819

[B50] Sohal V. S. Rubenstein J. L. R. (2019). Excitation-inhibition balance as a framework for investigating mechanisms in neuropsychiatric disorders. *Mol. Psychiatry* 24 1248–1257. 10.1038/s41380-019-0426-0 31089192 PMC6742424

[B51] Soltani Zangbar H. Ghadiri T. Seyedi Vafaee M. Ebrahimi Kalan A. Fallahi S. Ghorbani M. (2020). Theta oscillations through hippocampal/prefrontal pathway: importance in cognitive performances. *Brain Connect.* 10 157–169. 32264690 10.1089/brain.2019.0733

[B52] Tang Y. Zou H. Strong J. A. Cui Y. Xie Q. Zhao G. (2006). Paradoxical effects of very low dose MK-801. *Eur. J. Pharmacol.* 537 77–84. 10.1016/j.ejphar.2006.03.016 16626696

[B53] Torrisi S. A. Salomone S. Geraci F. Caraci F. Bucolo C. Drago F. (2017). Buspirone counteracts MK-801-induced schizophrenia-like phenotypes through dopamine D(3) receptor blockade. *Front. Pharmacol.* 8:710. 10.3389/fphar.2017.00710 29046641 PMC5632784

[B54] Tsai G. Coyle J. T. (2002). Glutamatergic mechanisms in schizophrenia. *Annu. Rev. Pharmacol. Toxicol.* 42 165–179. 10.1146/annurev.pharmtox.42.082701.160735 11807169

[B55] Valsamis B. Schmid S. (2011). Habituation and prepulse inhibition of acoustic startle in rodents. *J. Vis. Exp.* 55:e3446. 10.3791/3446 21912367 PMC3217252

[B56] van der Staay F. J. Rutten K. Erb C. Blokland A. (2011). Effects of the cognition impairer MK-801 on learning and memory in mice and rats. *Behav. Brain Res.* 220 215–229. 10.1016/j.bbr.2011.01.052 21310186

[B57] Wu J. Zou H. Strong J. A. Yu J. Zhou X. Xie Q. (2005). Bimodal effects of MK-801 on locomotion and stereotypy in C57BL/6 mice. *Psychopharmacology* 177 256–263. 10.1007/s00213-004-1944-1 15290006

