# Supplementary figures and images for: Behavioral features and disorganization of oscillatory activity in C57BL/6J mice after acute low dose MK-801 administration
Source: Front Neurosci. 2022 Sep 14;16:1001869. doi: 10.3389/fnins.2022.1001869 (PMC9515662; doi:10.3389/fnins.2022.1001869)

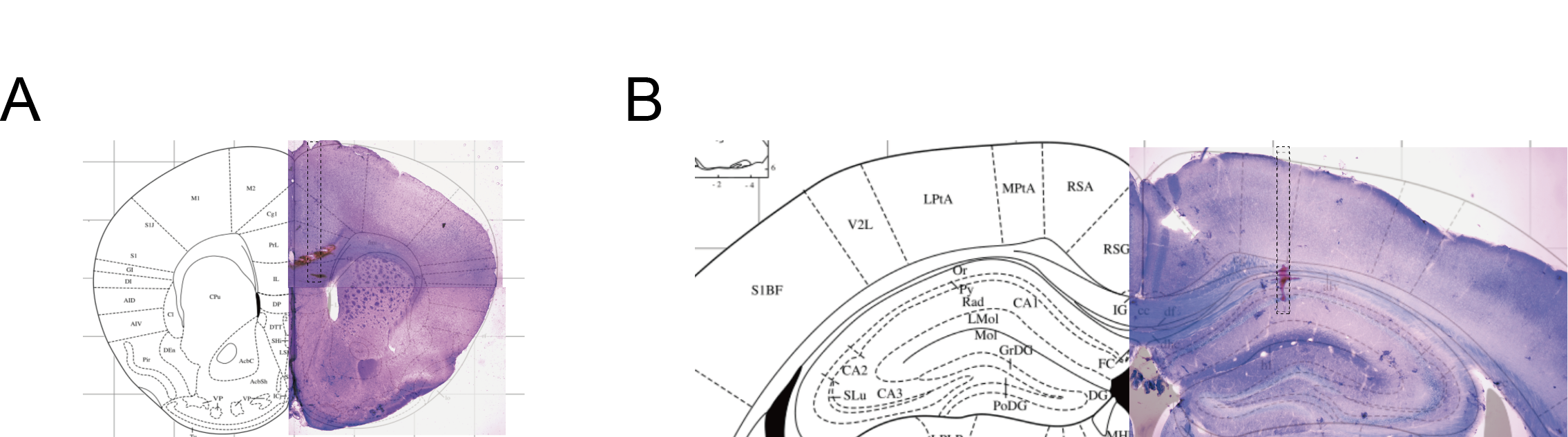

Supplement: Supplementary file 1 [file Image_1.TIF]
